# Additive effect of atropine eye drops and short-term retinal defocus on choroidal thickness in children with myopia

**DOI:** 10.1038/s41598-020-75342-9

**Published:** 2020-10-27

**Authors:** Samuel T.-H. Chiang, Philip R. K. Turnbull, John R. Phillips

**Affiliations:** 1 School of Optometry and Vision Science, The University of Auckland, Auckland, 1023 New Zealand; 2 Department of Medical Research, China Medical University Hospital, Taichung, Taiwan; 3 Department of Optometry, Asia University, Taichung, Taiwan

**Keywords:** Health care, Medical research, Paediatric research

## Abstract

Atropine eye drops and myopic retinal defocus each slow progression of myopia (short-sight). They also cause thickening of the choroid, and it has been suggested that the thickening is a precursor for reduced eye growth and slowed myopia progression. We investigated whether choroidal thickening due to optical defocus would add to thickening due to atropine when both were applied simultaneously. Addition would suggest that combining the two clinical treatments may improve efficacy of myopia control. We studied 20 children receiving 0.3% atropine daily for myopia control, over a period of 6 months. We imposed short periods of retinal defocus (1 h of myopic or hyperopic defocus (± 2.00D)) both before, and after 1 week and 3 and 6 months of atropine treatment. Prior to atropine, myopic or hyperopic defocus caused significantly thicker or thinner choroids respectively (± 12 µm, p < 0.001). After one week of atropine alone, thickness had increased (+ 21 µm; SD 17 µm; p < 0.001), and it increased further (by + 13 µm; SD 6 µm; p < 0.001) when exposed to myopic defocus. Atropine abolished choroidal thinning in response to hyperopic defocus. These effects remained the same after 3 and 6 months of atropine treatment. Our results show that additive effects of atropine and optical defocus are present at the level of the choroid, and suggest that combining optical and pharmaceutical treatments is likely to enhance efficacy of clinical myopia control.

## Introduction

Myopia (short-sight) is increasing in prevalence woldwide^1,2^ and has reached epidemic levels in East Asia, with 84% of school leavers in Taiwan^3^, and 96.5% of 19 year-old males in Seoul^4^ having myopia. Myopia results from abnormal enlargement of the eye, and as the sclera expands, the internal tissues of the eye, in particular the retina and choroid become stretched and damaged^5^. As a result, myopia is a significant risk factor for other sight-threatening conditions such as glaucoma, myopic maculopathy and retinal detachment, with the relative risk of developing these conditions increasing sharply with the degree of myopia^6^.

It is well-established in many animal species^7^ and in human studies^8^, that the refractive development of the eye is guided by the visual environment to which it is exposed. Specifically, eye growth is regulated by a visually guided emmetropisation process, which can detect the sign of retinal defocus and control eye growth to position the retina in the focal plane of the eye’s optics^7,9^. When the retina is exposed to hyperopic defocus (focal plane posterior to the retina) the choroid becomes thinner in a matter of minutes, both in chicks^10^ and in humans^11^. If the defocus is maintained over a much longer period (days or weeks), eye growth accelerates until the error of focus is minimized^12,13^. In contrast, myopic retinal defocus (focal plane anterior to the retina) causes rapid thickening of the choroid in chicks^10^, monkeys^14^ and humans^11^ and over time, slowing of eye growth^12,13^. Due to its location between the retina and sclera, it has been hypothesized that the choroid plays an intermediary role in the eye-growth signaling pathway from retina to sclera, with changes in choroidal thickness either reflecting or enacting changes in eye-growth^15,16^. Our understanding that eye growth can be manipulated by controlling the position of the focal plane relative to the retina, as summarized above, has led to the development of a range of optical methods for controlling the abnormal eye growth underlying myopia progression. Dual focus contact lenses^17,18^, multi-segment spectacle lenses^19^ and orthokeratology^20,21^ are used clinically^22^ to reduce eye growth and thus myopia progression, by imposing myopic defocus to parts of the retina to slow eye growth, while simultaneously providing clear foveal vision. However, there is also a well-tried and effective pharmaceutical method for inhibiting myopia progression using nightly instillation of atropine eye drops^23,24^. Although the way in which atropine works to reduce myopia progression is poorly understood, it is known that atropine, like myopic retinal defocus, also causes thickening of the choroid over a wide range of concentrations from 0.01^25^ to 1%^26^.

None of the optical or pharmaceutical methods for myopia control completely inhibit myopia progression^27^ and there is significant interest in determining whether combining therapies may increase overall treatment efficacy. Early clinical results indicate that the efficacy of orthokeratology in slowing myopia progression can be enhanced by concurrent use of atropine eye drops^28–30^.

This study investigates whether the choroidal thickening produced by simultaneous presentation of myopic defocus and atropine is greater than the thickness changes induced by atropine or optical defocus alone. A finding that the thickening effects of the two methods summate would be consistent with distinct mechanisms for optical and pharmaceutical methods of myopia control. However, regardless of mechanism, a finding of summation would imply that the efficacy of myopia control based on imposing myopic defocus to the retina could be increased by simultaneous administration of atropine.

## Methods

### Participants

Twenty Taiwanese children (9 male) aged between 6 and 14 years were recruited into the study through the Department of Ophthalmology, China Medical University Hospital, Taichung, Taiwan. Human Ethics approval was obtained from the Research Ethics Committee of China Medical University & Hospital (Protocol No./CMUHREC No: CMUH104-REC3-069) and the study was conducted in accordance with the Declaration of Helsinki. Informed consent was obtained from parents in writing. Informed assent was obtained from children verbally.

Participating children had been assessed by an Ophthalmologist and were preparing to start nightly atropine treatment to control myopia. The parent/guardian of each participant was given instructions on how to instil eye drops, and instructed to instil 1 drop of 0.3% atropine into both participant’s eyes at night. At the time of the study, 0.3% atropine was the concentration typically prescribed for myopia control at this study site. The mydriatic and cycloplegic effects of atropine were explained to parents and participants. At enrolment, the current spectacle correction was checked in all participants and upgraded to provide the full distance correction, if necessary. Participants were advised to continue wearing their spectacles as usual, and to remove them for close work if needed. They were also advised to wear sunglasses and a hat when outside, and the potential need for reading spectacles for near work was explained.

Inclusion criteria were: age 6–15 years at time of enrolment; Spherical Equivalent Refraction (SER = sphere + ½ cylinder) between − 0.75 D and − 4.50 D and visual acuity of at least 0.0 logMAR. Children with any known ocular pathology, astigmatism in either eye greater than − 1.00 DC, anisometropia greater than 1.00 D, or a history of myopia control (either pharmacological or optical methods) were excluded.

### Experimental design

The experimental eye of each participant was assigned to be either the dominant or non-dominant eye using permuted block randomisation, with a block size of four (https://www.sealedenvelope.com/help/redpill/latest/block/) to ensure equal numbers of dominant and non-dominant experimental eyes after every four participants were enrolled. Eye dominance was determined with a simple pointing task, and the assigned experimental eye for each participant remained the same throughout the study, with the contralateral eye acting as control.

**Figure 1:**
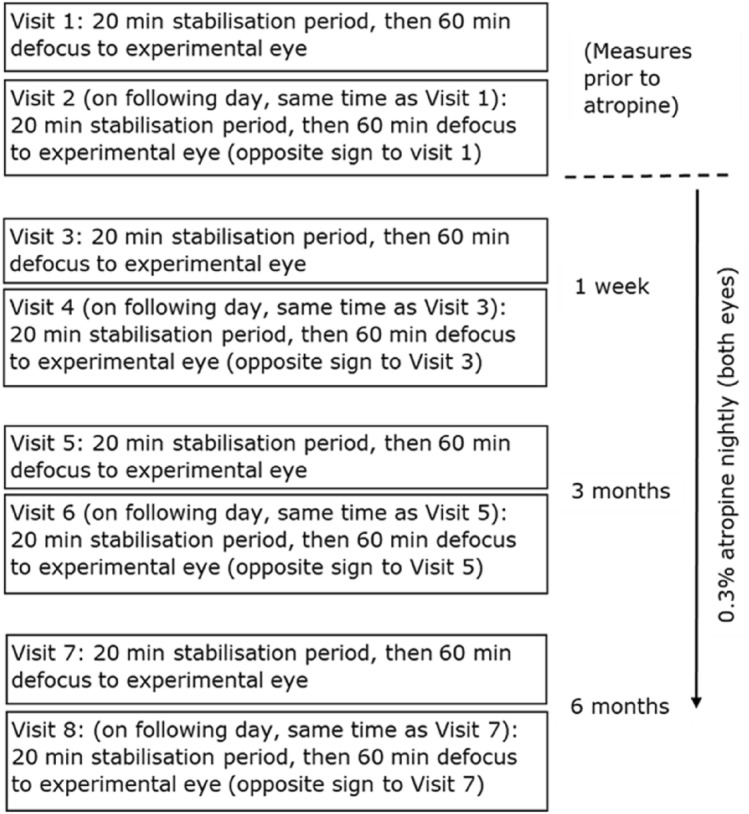
Summary diagram of measurement visits and atropine administration. Visits were in pairs, at the same time of day on successive days. The sign of defocus presented on the first day of a pair was randomly assigned and the sign of defocus presented on the second day was the opposite of the previous day (e.g. if a participant received myopic defocus at visit 1, then hyperopic defocus was presented at visit 2).

Outcome measures were made at four time points: before atropine, and after 1 week, 3 months and 6 months of atropine treatment. Measures for all subjects were completed between 1 and 6 pm on the relevant days. To minimise diurnal effects for each subject at each time point, measures were made over two visits at the same time of day (within one hour) on consecutive days (Fig. 1). At each visit, participants first viewed a video movie at 6 m for a 20-min stabilization period with full distance correction for both eyes, in order to reduce the influence of previous visual and non-visual tasks on choroidal thickness. Participants then viewed a video for 60 min at 6 m with both eyes fully corrected, but with either hyperopic or myopic retinal defocus (± 2.00 D) applied with a single focus ophthalmic spectacle lens to the experimental eye only. Optical Coherence Tomography (OCT) scans of the posterior eye (retina and choroid) in both eyes were made just prior to and at 20, 40 and 60 min during the 60-min video viewing period. Participants sat next to the OCT machine while viewing the video, so only a few seconds elapsed between video viewing and OCT measures. All OCT measures and video viewing was conducted under dim illumination (10 lx), as in previous studies^31^ to increase pupil size during measures prior to atropine use and thus improve the quality of OCT scans.

### Outcome measures

The primary outcome measure was change in sub-foveal choroidal thickness (SFCT), measured from Spectral Domain Optical Coherence Tomography images (Nidek RS-3000 RetinaScan Advance, Nidek, Japan). We followed the OCT protocols that we have previously used^11^, with the 6 mm “Macula Line” function centred on the fovea and ‘Choroidal’ scanning mode selected to obtain the cross-sectional chorio-retinal images. Images were exported as Bitmap files, and de-identified before measurements were made by masked observers. The inner and outer choroidal borders were independently identified and manually measured by the three masked investigators using ImageJ (https://imagej.nih.gov/ij/). Image dimensions were calibrated by embedding a line of known length on the Bitmap image, using the built in ‘measure’ function of the OCT software, giving an average scaling factor of 3.01 µm per pixel. Agreement between the three observers was assessed using Bland–Altman analysis^32^, giving a mean inter-observer difference of 1 µm (95% CI − 10 to + 9 μm) (see Supplementary Material, Figure S1). The choroidal thickness data presented in the results is derived from the average of the measures made on OCT images by the three masked observers.

Secondary outcome measures included axial eye dimensions (mean of 5 readings) measured using optical low coherence reflectometry (LenStar LS 900, Haag Streit AG, Switzerland) and refractive error (mean of 5 readings) measured using an open field autorefractor (Shin-Nippon Auto Refkeratometer NVision-K 5001 Japan). Baseline refractive error was measured without cycloplegia to avoid any effects of the cycloplegic agent that may have influenced choroidal thickness measures in previous studies ^33^. Measures of accommodation were made using the push-up method, and measures of pupil diameter were made with a pupil rule under normal room lighting (300–400 lx).

### Statistical analysis

Statistical analyses were performed using Matlab (2019a, Mathworks, USA). The choroidal thickness changes were measured as absolute changes from baseline of the relevant 60-min observation period. Pair-wise comparisons of choroidal thickness changes were compared with paired t-tests, using Bonferroni correction for multiple comparisons as required. A p-value < 0.05 was considered statistically significant.

## Results

Participant demographics and baseline measures of refraction and axial eye length for control and experimental eyes are shown in Supplementary Material Table S1. There were no significant differences between control and experimental eye parameters at Baseline. The baseline SFCT (prior to atropine or defocus) varied widely among participants (range, experimental eye: 166–469 µm, control eye: 156–434 µm) but there was no difference between mean baseline SFCT in experimental and control eyes (271 SD 72 µm vs 278 SD 74 µm, F_(1,38)_ = 0.08, p = 0.78). There was a significant relationship between the amount of myopia (mean spherical equivalent of both eyes) and the baseline SFCT (F_(18)_ = 4.48, p = 0.049), suggesting that choroidal thickness decreased by 32 µm per dioptre of myopia in these participants (Supplementary Material Figure S3).

### Effect of optical defocus on choroidal thickness prior to atropine

**Figure 2:**
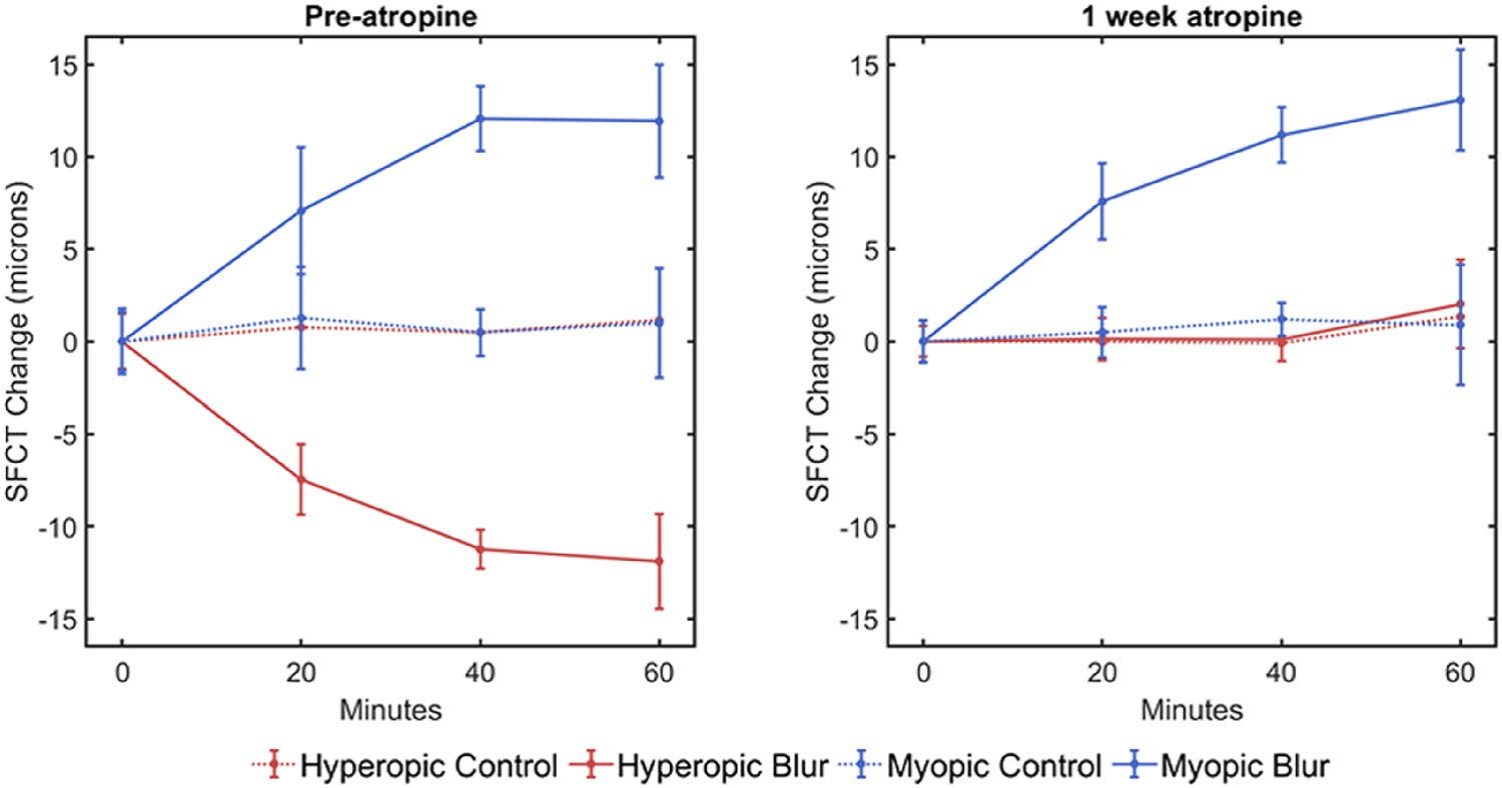
Changes in SFCT of the experimental eye after 60 min of continuous exposure to -2.00D of hyperopic (red) and + 2.00D of myopic (blue) defocus prior to atropine (left), then after 1 week of daily atropine to both eyes (right). Control eye responses shown as dotted lines. Prior to starting atropine, SFCT increased in response to myopic defocus, and decreased in response to hyperopic defocus. However, after daily atropine eye drops, the thinning response of SFCT when exposed to hyperopic defocus was abolished. In contrast, the thickening of SFCT seen when exposed to myopic defocus was unaffected by atropine (solid blue lines). Error bars represent ± one standard deviation.

Prior to atropine, 60 min of + 2.00D myopic defocus imposed in the experimental eye caused an increase in SFCT (+ 11.9 SD 6.96 µm), compared to the control eye (+ 1.0 SD 3.97 µm, t_(38)_ = 6.10, p < 0.001; Fig. 2 left, blue). In contrast, 60 min of -2.00D hyperopic defocus caused significant thinning of SFCT (− 11.9 SD 7.81 µm) compared to the control eye (+ 1.1 SD 4.10 µm, t_(38)_ = 6.59, p < 0.001, Fig. 2 left, red).

### Effect of 1 week of atropine on choroidal thickness and response to defocus

Measures of SFCT at baseline (prior to any defocus) were compared between the initial pre-atropine visit and the first visit following one week of atropine. Atropine alone increased SFCT in experimental eyes by a mean of + 21.0 SD 16.52 µm (t_(19)_ = 5.70, p < 0.001). After one week of atropine to both eyes, 60 min of imposed myopic defocus to the experimental eye caused choroidal thickening in the experimental eye (+ 13.1 SD 6.2 µm) compared to the control eye (+ 0.9 SD 3.46 µm, t_(38)_ = 7.66, p < 0.001; Fig. 2 right, blue). This additional choroidal thickening with myopic defocus following atropine was not different to the thickening in response to myopic defocus recorded prior to starting atropine (+ 11.92 SD 6.96 µm, t_(38)_ = 0.566, p = 0.575). However, following atropine, hyperopic defocus applied in the experimental eye no longer caused a decrease in SFCT compared to the control eye (experimental eye: + 2.0 SD 4.68 µm, control eye: + 1.3 SD 2.57 µm, t_(38)_ = 0.586, p = 0.561).

### Effects of atropine and defocus were independent of baseline SFCT

**Figure 3:**
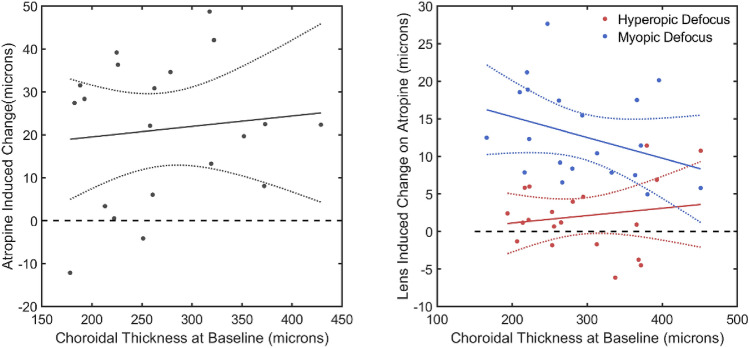
Left: Mean-difference plot of the change in SFCT with atropine versus SFCT in the experiment eye of each participant. (i.e. SFCT after one week of atropine minus SFCT prior to starting atropine) versus SFCT. SFCT increased by a mean of 21.0 SD 16.52 µm (t_(19)_ = 5.70, p < 0.001) after a week of atropine, and there was no relationship with baseline SFCT (r = 0.010, p = 0.673. Right: Mean-difference plots of changes in SFCT after 60 min of myopic (blue) and hyperopic (red) defocus following one week of daily atropine. There was no significant difference in the defocus-induced change in SFCT across the range of baseline choroidal thickness (Myopic defocus: r = 0.021, p = 0.536, Hyperopic defocus: r = 0.104, p = 0.166). Dotted lines represent the 95% confidence interval of the fitted regression.

Despite the wide range of baseline SFCT among participants, there was no significant difference in the magnitude of the increase in SFCT produced by atropine across this wide range of SFCT (Fig. 3 left, F = 0.54, p = 0.472).

Following one week of atropine the mean choroidal thickening associated with 60 min of sustained myopic defocus was + 13.1 SD 6.20 µm (t_(19)_ = 9.42, p < 0.001), but this thickening was not significantly associated with the baseline SFCT (F = 2.08, p = 0.166; Fig. 3 right). Similarly, after one week of atropine, there was no longer any choroidal thinning following 60 min of hyperopic defocus (+ 2.0 SD 4.68 µm, t_(19)_ = 1.93, p = 0.068), and there was no difference in the change in thickness across the range of baseline choroidal thickness (F = 0.358, p = 0.557; Fig. 3 right).

### Effect of atropine after 3 and 6 months

**Figure 4:**
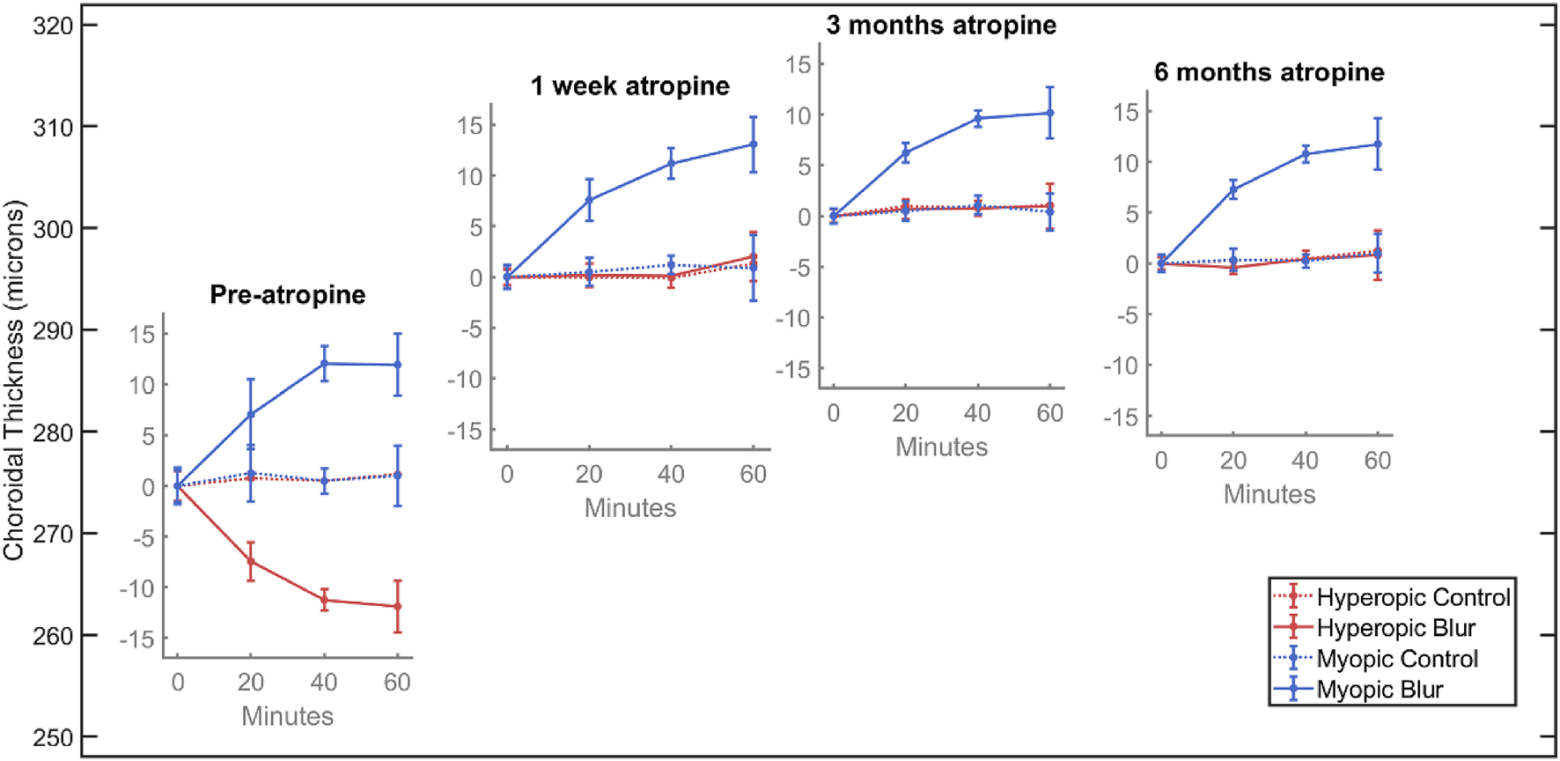
Graphical illustration of changes in SFCT during the 6 months of the study. The inset plots are positioned to show the mean SFCT at the first measurement (i.e. prior to defocus) at each visit. After the first pre-atropine visit, there is an increase of approximately 20 microns while on atropine, which remains approximately consistent at the 1-week, 3-month, and 6-month visits. Each inset plot shows the change in choroidal thickness in response to lens defocus at each visit. Each subplot shows that while on atropine, the choroid thickens in response to myopic defocus by approximately the same amount as prior to atropine, indicating that the thickening effect of myopic defocus is superimposed on the thickening effect of atropine. Further, it also shows that the choroidal thinning seen in response to hyperopic defocus is abolished while on atropine and this effect persists for all three visits up to six months.

After 3 and 6 months of nightly atropine, SFCT remained thicker than it was prior to atropine, and the increased thickness was similar to that at 1 week (mean of experimental and control eyes: 1 week: + 19.6 SD 13.51 µm, 3 months: + 25.9 SD 18.0 µm and 6 months: + 20.9 SD 19.28 µm thicker than baseline SFCT). Also, the choroidal thickening in response to myopic defocus seen prior to atropine and after one week of atropine was similar at both 3 and 6 months (3 months: experimental eye: + 10.2 SD 5.80 µm, control eye: + 0.4 SD 1.81 µm, t_(38)_ = 7.213, p < 0.001, and 6 months: experimental eye: + 11.8 SD 5.72 µm, control eye: + 1.0 SD 1.92 µm, t_(38)_ = 8.005, p < 0.001; Fig. 4 blue lines). The selective abolition of choroidal thinning in response to hyperopic defocus was also present after 3 and 6 months of nightly atropine (3 months: experimental eye: + 1.0 SD 2.19 µm, control eye: + 1.1 SD 1.61 µm, t_(38)_ = 0.164, p = 0.870, and 6 months: experimental eye: + 0.80 SD 2.16 µm, control eye: + 1.2 SD 2.02 µm, t_(38)_ = 0.605, p = 0.549; Fig. 4, red lines).

### Secondary outcomes

Changes in ocular biometry over the course of the study are shown in Table S2. In summary, there was a significant increase in anterior chamber depth and significant decrease in Lens thickness at 3 and 6 months compared to baseline as would be expected with atropine relaxing the ciliary muscle and flattening the lens. Vitreous chamber depth was significantly reduced at 3 months, but not at 6 months compared to baseline, and axial eye length increased significantly between 3 and 6 months.

Changes in refraction, amplitude of accommodation and pupil size during the study are shown in Table S3. In summary, refractions became significantly less myopic at one week compared to baseline (mean change + 0.20 SD 0.09D, t = 9.80, p < 0.001), but then became progressively more myopic, until at 6 months the mean refraction had returned to the baseline value (Table S3 and Figure S2). As a result, no participants needed a change to their glasses prescription during the 6 months of the study.

The mean binocular amplitude of accommodation at commencement of the study was + 17.5 SD 1.30 D, which reduced significantly after one week of atropine administration to + 1.37 SD 0.20 D and then remained unchanged for the 6 months of the study (Amplitude at 3 months: + 1.36 SD 0.20 D and at 6 months: + 1.36 SD 0.23 D) as shown in Supplementary Material Table S3 and Figure S3. The pupil size (mean of both eyes) at commencement of the study was + 4.08 SD 0.47 mm, which increased significantly after one week of atropine administration to 7.44 SD 0.38 mm and then remained unchanged for the 6 months of the study (pupil size at 3 months: 7.53 SD 0.37 mm and at 6 months: 7.55 SD 0.34 mm) as shown in Supplementary Material Table S3 and Figure S3.

## Discussion

This study investigated the independent and combined effects of atropine eye drops and short-term retinal defocus on the thickness of the choroid in children. With regard to defocus, our results are in line with previous human studies showing that imposition of myopic and hyperopic retinal defocus leads to choroidal thickening and thinning respectively within 60 min of applying defocus in adults^11, 31,34,35^ and children^33^. With regard to atropine, our results also corroborate previous findings that atropine eye drops themselves increase the thickness of the choroid in young adults ^25^ and children^26^. Our study also extends the finding in young adults that atropine abolishes the choroidal thinning induced by hyperopic retinal defocus^25,36^ and shows that this effect is also present in children susceptible to progressive myopia. The main new finding of our study is that short-term myopic defocus causes additional choroidal thickening in children whose choroidal thickness has already been increased by nightly treatment with atropine. Moreover, we also show that the choroidal thickening effect of nightly atropine remains constant over six months on atropine, as does the additive effect of short-term myopic defocus. Nightly atropine for 6 months also continues to inhibit the choroidal thinning in response to short-term hyperopic defocus that is seen without atropine. Such persistent effects of myopia control therapy on the choroid has also been reported with orthokeratology treatment, in which the subfoveal choroid increases in thickness by 16 µm at one month and remains unchanged at 12 months of treatment^37^.

A recent study of high concentration (1%) atropine in children, reported an increase in SFCT of + 24 μm after one week of treatment^26^. This suggests that in practice, the maximum subfoveal choroidal thickening achievable with atropine in children is approximately + 25 µm. Therefore, the increase in SFCT seen in the current study using 0.3% atropine (+ 21 µm at 1 week, + 26 µm at 3 months and + 21 µm at 6 months) is likely close to the maximum achievable with atropine alone. Consequently, our finding that the choroidal thickening induced by myopic defocus (+ 12 µm) simply added to the near-maximum thickening associated with atropine, strongly suggests that the upstream mechanisms underlying the two responses are distinct. Furthermore, the finding that the increase in SFCT with myopic defocus after atropine (+ 13 µm), was not different to that found prior to atropine (+ 12 µm) also suggests that two separate upstream mechanisms are involved.

We found considerable variation in the baseline choroidal thickness among subjects. However, both the changes in choroidal thickness produced with atropine and with defocus, were independent of baseline choroidal thickness—something we have also observed in presbyopic subjects^31^. This lack of correlation between changes in choroidal thickness and baseline choroidal thickness could occur if the changes in thickness take place in a sub-layer of the choroid, having a more constant thickness among participants than the full choroidal thickness. Such sub-layer specific changes have been reported previously: for example, choroidal thinning associated with ageing is mostly accounted for by thinning of Haller's layer^38^.

Our study confirms that atropine abolishes the choroidal thinning induced by hyperopic retinal defocus in children, as reported in previous studies of young adults^25,36^, without abolishing the thickening effect of myopic defocus. This finding is consistent with the suggestion by Nickla et al^39^ that in the chick model of myopia, choroidal thinning and thickening are mediated by different mechanisms. Nickla and colleagues proposed that choroidal thinning is mediated by contraction of choroidal, nonvascular smooth muscle, stimulated by acetylcholine, whereas choroidal thickening is mediated via a dopaminergic or nitrergic pathway. On this basis, atropine might be expected to block choroidal thinning independently from choroidal thickening.

In practical terms, our results show that atropine could block the choroidal thinning associated with hyperopic defocus resulting from the significant lag of accommodation associated with short working distances^40^, and therefore may provide additional benefit in children who habitually adopt very close working distances^41,42^.

During the 6-month study period, none of the children reported any significant clinical adverse event, with no reports of ocular irritation, allergic conjunctivitis or allergic dermatitis of the eyelid. The well-known side-effects of atropine administration were reported by some participants. Five participants reported significant sensitivity to light with atropine use: these were managed with sunglasses for outdoor activities. Most children managed the reduction in accommodation associated with atropine use by removing their distance correction for near tasks. Nine participants were provided with a reading correction for near work. This was expected for children with lower degrees of myopia, as removing their spectacles did not adequately compensate for the near demand of reading.

There are several limitations to our study. Current clinical practice has generally moved away from the earlier practice of prescribing higher concentrations of atropine (0.5–1%) to the use of low-dose atropine, typically 0.01–0.05% ^43,44^. Our study used 0.3% atropine, and so the effects we observed may be more apparent and rapid than that expected with low-dose atropine. Our study also only included Taiwanese children, and a relatively small sample (20), which may make it difficult to extrapolate to a more general population. Moreover, we used only dim light during video viewing, and it is possible that effects would differ under bright light conditions. While we controlled room lighting at 10 lx, as used in other studies measuring choroidal thickness changes in humans^11,45^, as the children were watching a DVD, there would have been temporal variation in retinal illumination, which can affect choroidal thickness^46^. Furthermore, although our results demonstrate superposition of the effects of atropine and optical defocus on choroidal thickness changes, whether this translates to a summative effect of their respective myopia-inhibiting effects remains to be seen. The assumption that these choroidal thickness changes are a precursor or biomarker for altered eye growth appears to be valid^15^ but has not been fully verified.

Nevertheless, due to the increasing worldwide prevalence of myopia, there is a pressing need to increase the efficacy of myopia control treatments. Our study shows that atropine combined with optical defocus causes the increase in choroidal thickness associated with each method to sum, giving a larger increase in choroidal thickness than either method alone. This suggests that combining optical and pharmaceutical treatments as a dual therapy is likely to enhance myopia control efficacy, which is consistent with the preliminary results of clinical studies (e.g. combining atropine with orthokeratology^28–30^).

## Supplementary information

Supplementary Information.

## Data Availability

The datasets generated during the current study are available from the corresponding author on reasonable request.
