# Supplementary material for: Additive effect of atropine eye drops and short-term retinal defocus on choroidal thickness in children with myopia
Source: Sci Rep. 2020 Oct 27;10:18310. doi: 10.1038/s41598-020-75342-9 (PMC7591535; doi:10.1038/s41598-020-75342-9)
Supplement: Supplementary file 1 — Supplementary Information. [file 41598_2020_75342_MOESM1_ESM.docx]

Supplementary Material

**Additive effect of atropine eye drops and short-term retinal defocus on choroidal thickness in children with myopia**

Samuel T-H, Chiang^1,2,3^, Philip R. K. Turnbull^1^, John R. Phillips^1,3^*

^1^ School of Optometry and Vision Science, The University of Auckland, New Zealand

^2^ Department of Medical Research, China Medical University Hospital, Taichung, Taiwan

^3^ Department of Optometry, Asia University, Taichung, Taiwan

**Inter-observer agreement**

Since the inner and outer boundaries of the choroid were determined manually by three masked observers, it was of interest to evaluate the repeatability between observers in determining choroidal thickness. Repeatability of the measures between observers was assessed by Bland-Altman analysis (Bland & Altman, 1999) and Intra-class correlation coefficient (ICC).

For the Bland-Altman analysis, the mean inter-observer difference and 95% limits of agreement were as follows: A vs B: -1 ± 5 μm (95% CI -11 to +8 μm); A vs C: -1 ± 5 μm (95% CI -10 to +9 μm); B vs C: 0 ± 5 μm (95% CI -9 to +10 μm), see *Figure 6* below for the Bland-Altman plot analyses for the three comparisons. The ICC analysis showed a very high correlation coefficient of 0.99 between the three observers.

| (a) | (b) |
| --- | --- |
| (c) |  |

***Figure S1.*** *Bland-Altman plots of inter-observer agreement on subfoveal choroidal thickness measurements between observers. (a) observer A vs B. (b) observer A vs C. (c) observer B vs C.*

**Participants demographics and Baseline data**

Details of the participants and baseline measures for Control and Experimental eyes are shown in Table S1. There were no significant differences between Control and Experimental eye parameters at Baseline.

| **Gender** | Male | 9 | 45% |  |
| --- | --- | --- | --- | --- |
|  | Female | 11 | 55% |  |
| **Ethnicity** | Taiwanese | 20 | 100% |  |
| **Allocation to**  **Exp eye** | Dominant eye | 10 | 50% |  |
|  | Non-dominant eye | 10 | 50% |  |
| **Age**  **(Years)** | Male | 9.89 ± 2.62 |  | Whole group  8.95 ± 2.31 |
|  | Female | 8.18 ± 1.78 |  |  |
| **Mean Sphere**  **(SER, Dioptres)** | Control eye | -1.64± 0.95 | p = 0.76 | Whole group  −1.63 ± 0.90 |
|  | Experimental eye | - 1.61 ± 0.90 |  |  |
| **Axial Length (mm)** | Control Eye | 24.12 ± 1.19 | p = 0.81 | Whole group 24.13 ± 1.17 |
|  | Experimental eye | 24.14 ± 1.17 |  |  |
| **Accommodation**  **(AoA, Dioptres)** | Control eye | 17.55± 1.47 | p = 0.87 | Whole group  17.53 ± 1.30 |
|  | Experimental eye | 17.50± 1.47 |  |  |
| **Pupil Size**  **(mm)** | Control eye | 4.08± 0.54 | p = 0.99 | Whole group  4.08 ± 0.47 |
|  | Experimental eye | 4.08± 0.47 |  |  |

***Table S1:*** *Baseline data for the 20 included participants (9 Males and 11 Females). Gender, ethnicity, eye allocation, age, spherical equivalent refraction (SER, D), Axial Eye Length (mm), amplitude of accommodation (AoA, D) and pupil size (mm).*

**Secondary Outcomes**

Table S2 shows the mean ocular biometry measures (central corneal thickness, axial eye length etc.) as they changed from the baseline measures prior to atropine (time 0) to 3 and 6 months of atropine use. In summary, there was a significant increase in anterior chamber depth and significant decrease in Lens thickness at 3 and 6 months compared to baseline. This is expected and likely the result of atropine relaxing the ciliary muscle and flattening the lens. Vitreous chamber depth was significantly reduced at 3 months, but not at 6 months and axial eye length increased significantly between 3 and 6 months.

|  |  | Control Eye | | Experimental Eye | | Mean | | Absolute Mean Change | | |
| --- | --- | --- | --- | --- | --- | --- | --- | --- | --- | --- |
|  | Month | Mean | SD | Mean | SD | Mean | SD | Mean | SD | P |
| CCT (µm) | 0 | 552.05 | 22.70 | 551.45 | 23.58 | 551.75 | 22.96 | 0.00 | 0.00 |  |
|  | 3 | 552.30 | 24.05 | 553.75 | 24.82 | 553.03 | 24.23 | 1.28 | 4.39 | 0.22 |
|  | 6 | 553.30 | 23.33 | 554.75 | 25.83 | 554.03 | 24.38 | 2.28 | 5.56 | 0.10 |
| AD (mm) | 0 | 3.16 | 0.34 | 3.15 | 0.33 | 3.15 | 0.34 | 0.00 | 0.00 |  |
|  | 3 | 3.27 | 0.31 | 3.25 | 0.33 | 3.26 | 0.32 | 0.11 | 0.09 | **<0.001** |
|  | 6 | 3.27 | 0.32 | 3.24 | 0.32 | 3.25 | 0.32 | 0.10 | 0.09 | **<0.001** |
| LT (mm) | 0 | 3.43 | 0.19 | 3.43 | 0.17 | 3.43 | 0.18 | 0.00 | 0.00 |  |
|  | 3 | 3.36 | 0.14 | 3.39 | 0.17 | 3.37 | 0.15 | -0.06 | 0.08 | **0.029** |
|  | 6 | 3.37 | 0.16 | 3.37 | 0.16 | 3.37 | 0.16 | -0.06 | 0.13 | **0.036** |
| VCD (mm) | 0 | 17.07 | 1.09 | 17.11 | 1.05 | 17.09 | 1.05 | 0.00 | 0.00 |  |
|  | 3 | 17.02 | 1.11 | 17.06 | 1.09 | 17.04 | 1.09 | -0.05 | 0.08 | **0.026** |
|  | 6 | 17.10 | 1.06 | 17.15 | 1.03 | 17.12 | 1.03 | 0.03 | 0.05 | 0.06 |
| AL (mm) | 0 | 24.12 | 1.19 | 24.14 | 1.17 | 24.13 | 1.17 | 0.00 | 0.00 |  |
|  | 3 | 24.12 | 1.19 | 24.15 | 1.17 | 24.13 | 1.17 | 0.00 | 0.06 | 0.86 |
|  | 6 | 24.20 | 1.17 | 24.21 | 1.16 | 24.20 | 1.15 | 0.07 | 0.06 | **<0.001** |

***Table S2.*** *Summary table for ocular biometry components made by LenStar reflectometer measurement. Central Corneal Thickness (CCT), Anterior Chamber Depth (AD), Thickness of the Crystalline Lens (LT), Vitreous Chamber Depth (VCD) and Axial length (AL). P-values were calculated with reference to baseline and Bonferroni corrected values < 0.05 are in bold.*

Table S3 shows the changes in refraction, amplitude of accommodation and pupil size from baseline (prior to atropine and at 1 week, 3 months and 6 months of atropine use. In summary, refractions became significantly less myopic at one week compared to baseline (mean change 0.20 ± 0.09D), but then became progressively more myopic, until at 6 months the mean refraction had returned to the baseline value (Table S3 and Figure S2)

|  |  | Control Eye | | Experimental Eye | | Mean | | Absolute Mean Change | | |
| --- | --- | --- | --- | --- | --- | --- | --- | --- | --- | --- |
|  | Visit | Mean | SD | Mean | SD | Mean | SD | Mean | SD | P |
| Refraction (D) | 0W | -1.64 | 0.95 | -1.61 | 0.90 | -1.63 | 0.90 |  |  |  |
|  | 1W | -1.41 | 0.95 | -1.44 | 0.89 | -1.43 | 0.90 | 0.20 | 0.09 | **<0.001** |
|  | 3M | -1.49 | 0.97 | -1.48 | 0.93 | -1.48 | 0.92 | 0.14 | 0.15 | **0.002** |
|  | 6M | -1.64 | 0.95 | -1.62 | 0.93 | -1.63 | 0.90 | 0.00 | 0.21 | 0.95 |
| AoA (D) | 0W | 17.55 | 1.47 | 17.50 | 1.47 | 17.53 | 1.30 |  |  |  |
|  | 1W | 1.33 | 0.16 | 1.39 | 0.27 | 1.37 | 0.20 | -16.16 | 1.28 | **<0.001** |
|  | 3M | 1.34 | 0.21 | 1.38 | 0.22 | 1.36 | 0.20 | -16.17 | 1.23 | **<0.001** |
|  | 6M | 1.37 | 0.23 | 1.34 | 0.31 | 1.36 | 0.23 | -16.16 | 1.22 | **<0.001** |
| Pupil (mm) | 0W | 4.08 | 0.54 | 4.08 | 0.47 | 4.08 | 0.47 |  |  |  |
|  | 1W | 7.43 | 0.41 | 7.45 | 0.46 | 7.44 | 0.38 | 3.36 | 0.64 | **<0.001** |
|  | 3M | 7.58 | 0.47 | 7.48 | 0.47 | 7.53 | 0.37 | 3.45 | 0.54 | **<0.001** |
|  | 6M | 7.58 | 0.44 | 7.53 | 0.38 | 7.55 | 0.34 | 3.48 | 0.63 | **<0.001** |

*Table S3. Summary of the refraction, amplitude of accommodation and pupil size of the Control and Experimental eyes and their mean, at each stage of the study. 0W= 0-week (Baseline), 1W = 1 week, 3M = 3 months and 6M = 6 months. P-values were calculated with reference to baseline and Bonferroni corrected values ≤ 0.05 are in bold.*


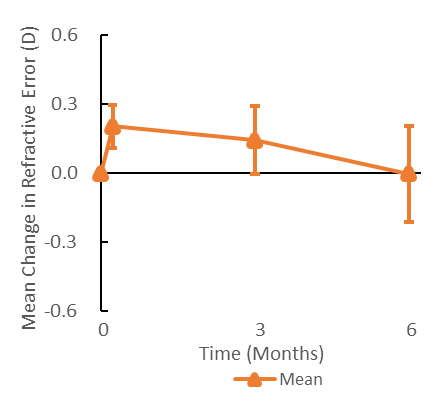


***Figure S2:*** *Mean changes in children’s refractive error over the 6-month study period. Mean of Control and Experimental eyes Error bars show ± 1 SD.*

In agreement with previous studies, we found that children with higher levels of myopia tended to have thinner choroids (Figure S3 left), and that atropine exerted unwanted effects on the eye including a reduction in amplitude of accommodation and an increase in pupil diameter (Figure S3 right).


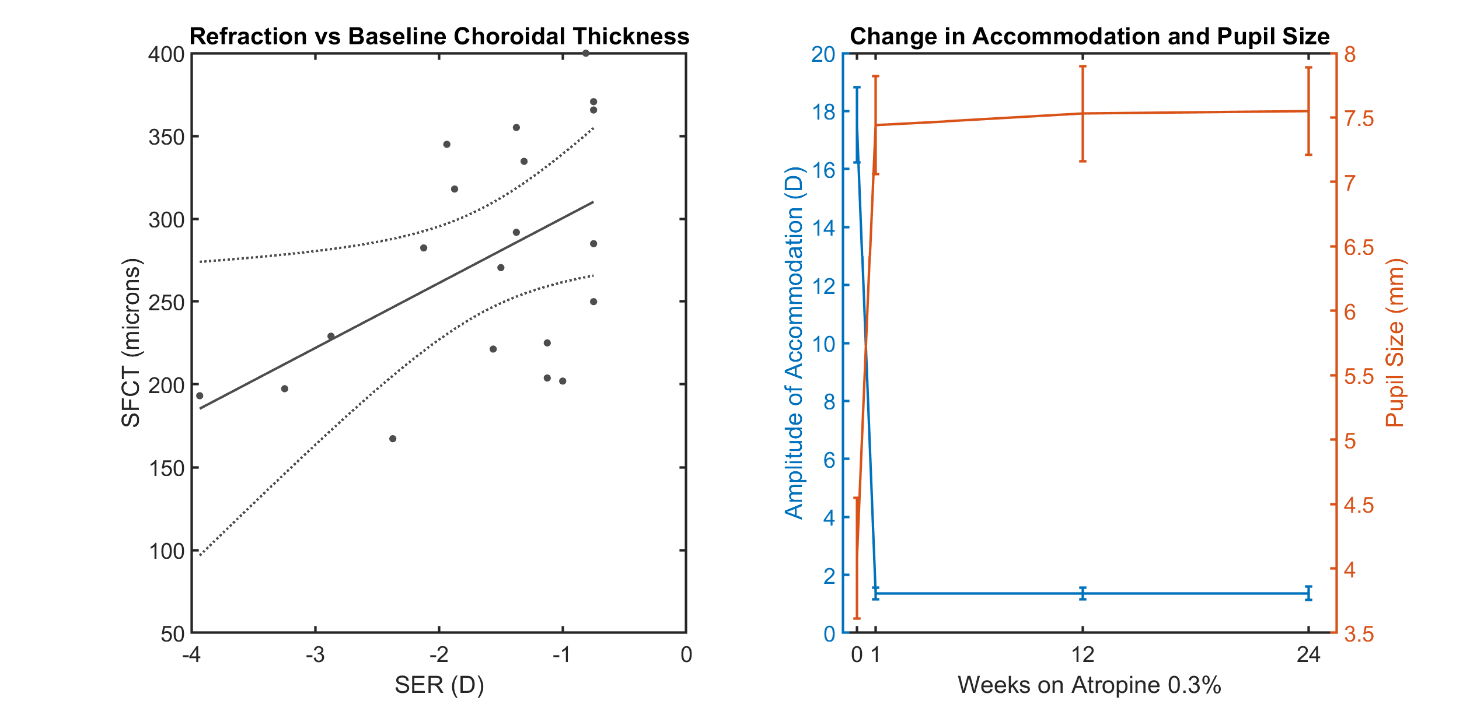


***Figure S3.*** *Left: There was a significant relationship between the amount of myopia (mean spherical equivalent of both eyes) and the baseline measurement of subfoveal choroidal thickness (F(18) = 4.48, p = 0.049). Right: After starting atropine 0.3% nightly, there was a significant decrease in the amplitude of accommodation from 17.5D at baseline to 1.4D at subsequent visits. Similarly, pupil diameter increased from 4.1mm at baseline to approximately 7.5mm at later visits, while on atropine.*
